# Dr. L. C. Fischer, Atlanta Surgical Clinic, at the Davis & Fischer Sanatorium

**Published:** 1913-12

**Authors:** 


### DR. L. C. FISCHER, ATLANTA SURGICAL CLINIC, AT THE DAVIS & FISCHER SANATORIUM.

The first case shown and operated upon was a goitre. History: The patient, a school girl 17 years of age, well developed and apparently in good health, came to me complaining that she had a choking sensation and that she could not get her breath good; that she suffered shortness of breath all the time and that she was very nervous. She has had the usual diseases of childhood, had Typhoid fever six years ago, ill for nine weeks. Has been of nervous temperament all of her life, that the nervousness increased with the appearance of menstruation, which appeared at or about the thirteenth year. That the nervousness was worse at this time than in the interval. For the last year she has noticed that she has been much more nervous than heretofore, she has had an enlargement in the throat or neck. She noticed this enlargement has grown more for the last few months, that when she was given medicine it apparently diminished in size, up to the last month, when it began to grow rapidly. Her pulse became fast, shortness of breath and nervousness increased markedly up to the time she consulted me on Nov. 1st, 1913. Her family history is negative, except that her father died of Phthesis some months ago. She has five sisters and one brother, all well and strong, mother living and in good health.

Upon examination I found the Thyroid gland much enlarged, as you will see more on the right side, breathing was difficult, temperature normal, pulse 140, blood pressure 85. The gland is freely movable and soft, my diagnosis is a large celloid goitre. There is no evidence of Basedow's disease, (or exopthalmic goitre).

After making the ordinary preparations for operation, with the patient in position, with shoulders elevated and the head thrown slightly back, an ordinary Kocher's incision is made through the skin and the Platisma Muscle, catching the superficial jugular veins on each side, this brings you down to the sternohyoid and sterno-thyroid muscles which are easily separated. Often it is necessary to cut these, but in this case they are easily retracted to one side which exposes the thyroid gland in its capsule, which is formed by the deep cervical fascia. Incising and dissecting the sheath from the gland, it is bound down tightly on its inner side, the remainder of the right lobe is freely movable. The right lobe we will remove entirely up to the isthmus, first ligating the superior and inferior thyroid arteries, then ligating the isthmus, cut through the gland tissue removing the lobe. The left lobe is more easily removed, it is dissected up and found to be not so large as the right side, after ligating the arteries, as on the right side, I will remove about two-thirds of this lobe, taking care to leave that part of the gland in that is normally attached to the isthmus. In all goitre operations great care should be taken to leave at least one-sixth of the gland to prevent the complications that will most certainly appear if the entire gland is removed. These complications are idiocy, cretinism, disturbances of growth and the sexual functions, together with the cessation of the menstrual flow. I have recently seen two patients in which I thought too much of the gland had been removed, where the menstruation had not appeared since the operation. Both of the women were young and unmarried and did not give any symptoms of pregnancy; the administration of thyroid extract for sixty days restored the normal flow. I have been asked often to demonstrate the parathyroids, it is useless for me to add that I have never attempted this. The number of these glands vary greatly, most usually four, they are located well down outside of the sheath of the Thyroid gland, near its upper and lower poles. The removal of all or the majority of them may and often does result in tetany. After the removal of the greater part of the left lobe, first dissecting back from the anterior surface the true capsule, remove the portion of the gland desired, then with plain gut close the capsule up over the portion of the gland left in. Care should be taken not to close the tissues over the remaining portion of the gland too tight, as this will cause much distress to the patient besides often causing symptoms of hyperthyroidism. I will close the muscles back normally with plain gut catching the Platisma and the fascias in the same line of sutures with chromic or tanned gut, closing the skin with clips. Without infection you will get a beautiful scar, in a large majority of cases the scar will not be noticed after sixty days unless one knows to look for it. The incision is made directly in the fold of the skin of the neck where it often is not at all noticable, and unless one knows the difference will be taken for one of the normal folds of the skin.

Case 2. The second case is one of appendicitis, the patient is a child eight years old, his general health is good, has had one or two previous attacks. Two years ago he had an attack that his mother stated was something like cramp colic. At the time he had had some kind of a "nervous spell," he did not lose consciousness at any time but would awaken from sleep much excited, and even in the day time would have one or two of these spells. He has had the usual diseases of childhood, and has suffered for the last two years from indigestion. Family history negative, mother and father living, in good health, one sister, younger, living, health good. I was called to see him twenty-four hours ago. He complained of pain all over the abdomen, located especially over McBurney's point, had not been able to retain nourishment for the last twelve hours. He had been sick for more than thirty-six hours. Upon examination I found his temperature 100, pulse 110, respiration 22, tenderness all over the abdomen, but located especially in the right Iliac fossa, leucocyte count 22,500.

Operation: The patient has not been given any nourishment by mouth since he has been in my care, only an enema. One of the mistakes that we often make is to give a purgative in Appendicitis, this increases the peristalsis of the bowels, making it much more likely to have a rupture of the appendix than where the bowels are left alone. I have not treated other than surgically, a case of appendicitis in a great many years. It has been my misfortune to see several cases recently where they were so far advanced the operation was not advisable. I advise operation in appendicitis as soon as the diagnosis is made. In this way we know what is in the abdomen, otherwise there is nothing but a guess. My incision is made as near the anterior-superior spinous process as possible, this brings you down through a regular gridiron incision to the cecum at the junction of the ilium. At this point there is less tension to make on the gut to get the appendix up than at any other point. The upper portion of the cecum is bound down normally to the posterior abdominal wall. The appendix is taken off in tne usual way, inverting the stump. Some operators are leaving the appendix stump after ligating it and not inverting. I do not approve of this neither do I think it good surgery, the stump after being ligating is bound to be absorbed, this is left free in the peritoneal cavity and is liable to cause infection from the remaining portion of the mucous membrane of the stump, then too, you leave a raw surface which is liable to cause adhesions that will give the patient much pain and discomfort. In closing the Peritoneum I hold this well up closing peritoneal surface to peritoneal surface, this will prevent the possibility of adhesions. Close the muscles together with one chromic of two plain, using two chromics to close the aponeurosis of the external oblique. Closing the skin with clips. After closing all of my incisions I like them painted over with tincture of Iodine, about 7 per cent., and ordinary dressing is applied, it will not be changed for a week unless necessary. As a rule the patient is allowed to be up and about the Sanatorium in ten days.

